# Iron is increased in the brains of ageing mice lacking the neurofilament light gene

**DOI:** 10.1371/journal.pone.0224169

**Published:** 2019-10-23

**Authors:** James C. Vickers, Anna E. King, Graeme H. McCormack, Aidan D. Bindoff, Paul A. Adlard

**Affiliations:** 1 Wicking Dementia Research and Education Centre, University of Tasmania, Hobart, Australia; 2 The Florey Institute of Neuroscience and Mental Health, University of Melbourne, Melbourne, Australia; Nathan S Kline Institute, UNITED STATES

## Abstract

There has been strong interest in the role of metals in neurodegeneration, and how ageing may predispose the brain to related diseases such as Alzheimer’s disease. Recent work has also highlighted a potential interaction between different metal species and various components of the cytoskeletal network in the brain, which themselves have a reported role in age-related degenerative disease and other neurological disorders. Neurofilaments are one such class of intermediate filament protein that have a demonstrated capacity to bind and utilise cation species. In this study, we investigated the consequences of altering the neurofilamentous network on metal ion homeostasis by examining neurofilament light (NFL) gene knockout mice, relative to wildtype control animals, at adulthood (5 months of age) and advanced age (22 months). Inductively coupled plasma mass spectroscopy demonstrated that the concentrations of iron (Fe), copper (Cu) and zinc (Zn) varied across brain regions and peripheral nerve samples. Zn and Fe showed statistically significant interactions between genotype and age, as well as between genotype and region, and Cu demonstrated a genotype and region interaction. The most substantial difference between genotypes was found in Fe in the older animals, where, across many regions examined, there was elevated Fe in the NFL knockout mice. This data indicates a potential relationship between the neurofilamentous cytoskeleton and the processing and/or storage of Fe through ageing.

## Introduction

Divalent cation metal species such as zinc, iron and copper have important normal cellular functions in the nervous system, but may also be involved in pathological processes underlying neurodegenerative diseases such as Parkinson’s disease (PD), amyotrophic lateral sclerosis (ALS) and Alzheimer’s disease (AD) [1–3]. Indeed, metal ions have been implicated in many aspects of the AD cascade [4], including a potentially central role in the aggregation of the two primary pathological hallmarks of AD, the beta amyloid plaques and neurofibrillary tangles [5]. In this regard, major proteins implicated in neurodegenerative diseases such as the amyloid precursor protein (APP), tau and alpha-synuclein and tau have been shown to be involved in metal ion processing and transport [6–8]. Furthermore, metal ions may promote aggregation of these proteins, contributing to hallmark pathological features [9].

A range of brain proteins have the capacity to bind metal species, with major cell groups, such as neurons, microglia and astrocytes likely having a spectrum of roles in terms of the storage, metabolism and use of metals. Neurofilaments (NF) are major neuronal proteins and have putative roles in stabilising axons and regulating axonal diameter [10]. Particular subsets of neurons demonstrate high levels of the NF ‘triplet’ proteins (NF light (NFL), medium (NFM) and heavy (NFH) see [10] for review on subunit structure and function), and these correspond to subpopulations of neurons that are vulnerable to degeneration in AD (cortical pyramidal neurons), PD (substantia nigra compacta) and ALS (cortical, brainstem and spinal motor neurons) [11–13]. The NFM and NFH subunits have long tail domains that project from the intermediate filament backbone, and which contain glutamate-rich, negatively charged subregions. In this regard, these protein regions may bind to cations, with accessibility regulated by phosphorylation of the tail domains [14].

Neurofilament accumulation in abnormal (dystrophic) neurites associated with beta amyloid plaques in the cortex is one of the earliest neuronal changes in the sequence of pathological changes leading to AD [13]. The NFL protein is a requisite component for the NF triplet to form intermediate filaments in neurons. We have also recently demonstrated that the ablation of NFL in a transgenic mouse model of AD (APPswe/PS1dE9) results in increased beta amyloid plaque deposition [15]. These data suggest that the absence of NFs may have an early role in amyloid misprocessing. Furthermore, substantia nigra neurons in PD also lose NF content [11], which is associated with the accumulation of iron in these nerve cells [1]. Collectively, these data suggest that NF triplet-abundant neurons may show changes in these cytoskeletal proteins as part of the disease process of major neurodegenerative changes, and that such changes contribute to subsequent pathological alterations potentially with respect to a role in metal biology within neurons. The following study explores the potential consequences of a major perturbation of NFs with metal ions, on the background of ageing. We have utilised mass spectrometry techniques to compare the abundance of metal species implicated in major neurodegenerative diseases, such as iron (Fe), copper (Cu) and zinc (Zn), in the brains and peripheral nerves of NFL KO mice as compared to C57BL/6 mice at adulthood (5 months of age) and following ageing (22 months of age).

## Materials and methods

### Animals and tissue processing

All animal experimentation was approved by the University of Tasmania Animal Ethics Committee (Approval Nos A12780 and A15120), in accordance with the Australian code of practice for the care and use of animals for scientific purposes. NFL KO mice were obtained from the Nathan Kline Institute (Dr. Mala Rao) and were developed in the laboratory of Dr. Jean-Pierre Julien [16]. NFL KO mice were maintained as a homozygous knockout colony. NFL KO mice (on a C57BL/6 background strain) were compared with wild-type (WT) (C57BL/6) mice, the predominant background strain of the mice [16,17]. Animals were housed in standard conditions (20°C, 12/12 hours light/dark cycle), with at least 2 animals in each cage with access to food and water *ad libitum*. Cohort sizes were as follows, 5 months (n = 11 NFL-KO, n = 7 WT) and 22 months (n = 6 NFL-KO, n = 7 WT).

### Tissue collection and processing

Animals were anaesthetised with 140mg/Kg sodium pentobarbitone (intraperitoneal) and perfused transcardially with phosphate buffered saline for 3 mins to clear blood from the vasculature. Tissue was dissected under microscopic guidance. Briefly, cortical tissue, hippocampus, olfactory bulb and cerebellum were collected from each hemisphere and the right hemisphere used in analysis; brain stem tissue was collected and left and right sciatic nerves were pooled for analysis. Upon collection, tissue was rapidly snap frozen in liquid nitrogen and stored at -80°C until used. Tissue was freeze dried in 96 well plates for 48hrs at -80°C. Dried tissue was kept at room temperature until used.

### Inductively coupled plasma mass spectroscopy (ICPMS) analysis

Tissue samples were coded and processed for inductively coupled mass spectroscopy analysis at the Florey Institute of Neuroscience and Mental Health. Tissue samples were lyophilised and then digested with nitric acid (65% Suprapur, Merck) overnight, followed by heating at 90°C for 20 min using a heat block. Samples were then removed from the heat block and an equivalent volume of hydrogen peroxide (30% Aristar, BDH) added to each sample. Once samples had finished digesting, they were heated for a further 15 mins at 70°C. Samples were then diluted with 1% nitric acid diluent. Measurements were made using an Agilent 7700 series ICPMS instrument under routine multi-element operating conditions using a Helium Reaction Gas Cell. The instrument was calibrated using 0, 5, 10, 50, 100 and 500 ppb of certified multi-element ICPMS standard calibration solutions (ICP-MS-CAL2-1, ICP-MS-CAL-3 and ICP-MS-CAL-4, Accustandard) for a range of elements, and we also utilised a certified internal standard solution containing 200 ppb of Yttrium (Y89) as a control (ICP-MS-IS-MIX1-1, Accustandard).

### Statistical analysis

Mixed effects models were fitted using the lme4 package in R. Random intercepts for each subject were specified in order to account for non-independence between brain regions within animals. Model assumptions were checked using standard graphical techniques, and a *log*_*e*_-transformation applied to Zn and Fe variables to improve normality of residuals and homogeneity of error variance. One substantial outlier was removed from Cu. Type III F statistics and 95% Confidence Intervals for the estimated marginal means were computed using the Kenward-Roger approximation for degrees of freedom. A measure of standardized effect size, Cohen’s *f*^2^ was calculated for the effect of genotype for each metal using the formula, $f^{2}=\frac{R_{AB}^{2}-R_{A}^{2}}{1-R_{AB}^{2}}$ where $R_{AB}^{2}$ is the coefficient of determination for the full model, and $R_{A}^{2}$ is the coefficient of determination for a reduced model that does not contain the term of interest. The method of Nakagawa and Schielzeth [18] for computing a marginal pseudo-*R*^2^ for mixed models was used to calculate *R*^2^ coefficients.

## Results and discussion

**Fig 1:**
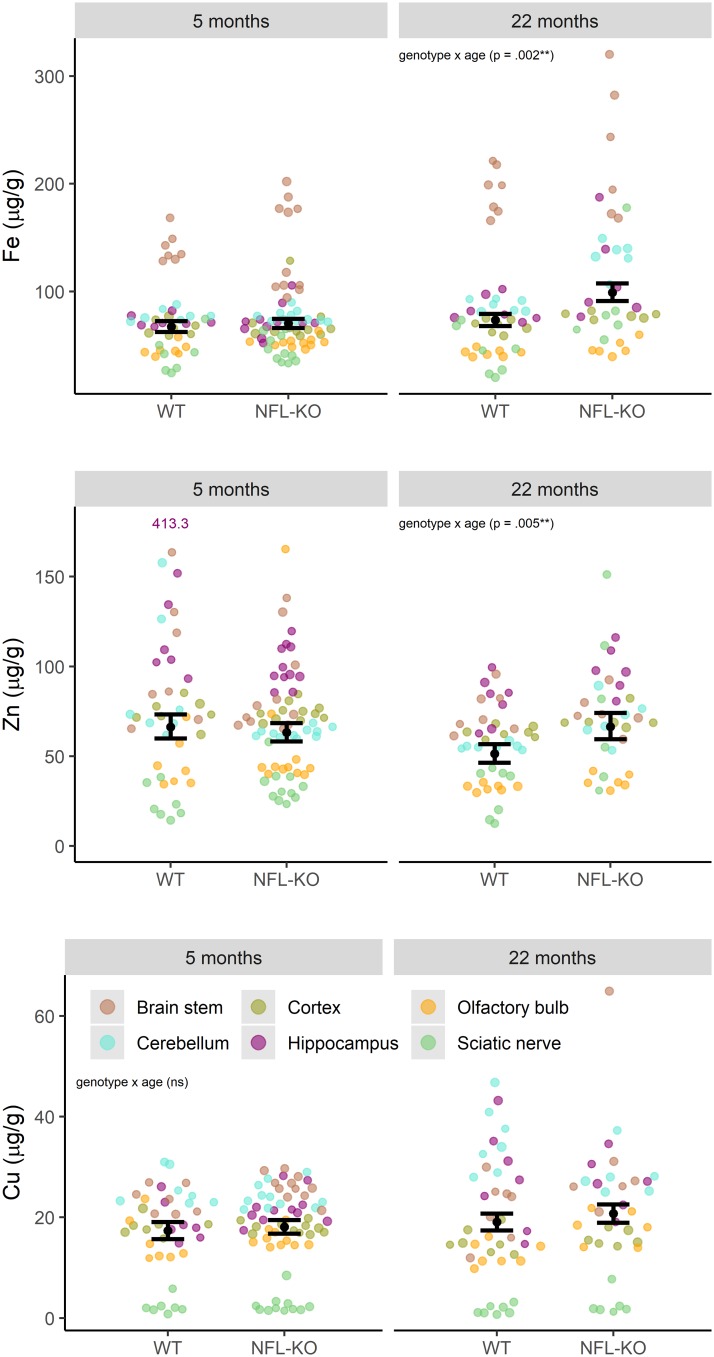
Metal composition at different ages. Fe, Zn, and Cu in sampled brain regions at 5mo and 22mo, for WT and NFL-KO mice. Error-bars show estimated marginal means (over all regions) and 95% CI, showing the strength and direction of these patterns of metal accumulation. There were significant genotype x age interactions for Zn and Fe (Zn: F_(1, 27)_ = 9, *p* = .005; Fe: F_(1, 27)_ = 11, *p* = .002).

Broadly, Fe content was increased in NFL KO and WT mice with ageing across most nervous system samples, with the exception of the olfactory bulb. In this regard, there was a more substantial ageing-related increase in Fe content in NFL KO mice relative WT animals, particularly in the cerebellum, cortex, hippocampus and sciatic nerve (Fig 1). For many CNS regions, there was higher content of Zn in older NFL KO mice relative to WT mice. In addition, the sciatic nerve of NFL KO mice showed higher levels of Zn that WT mice at both 5 and 22 months of age. No consistent pattern of difference between NFL KO and WT mice was observed for Cu.

**Fig 2:**
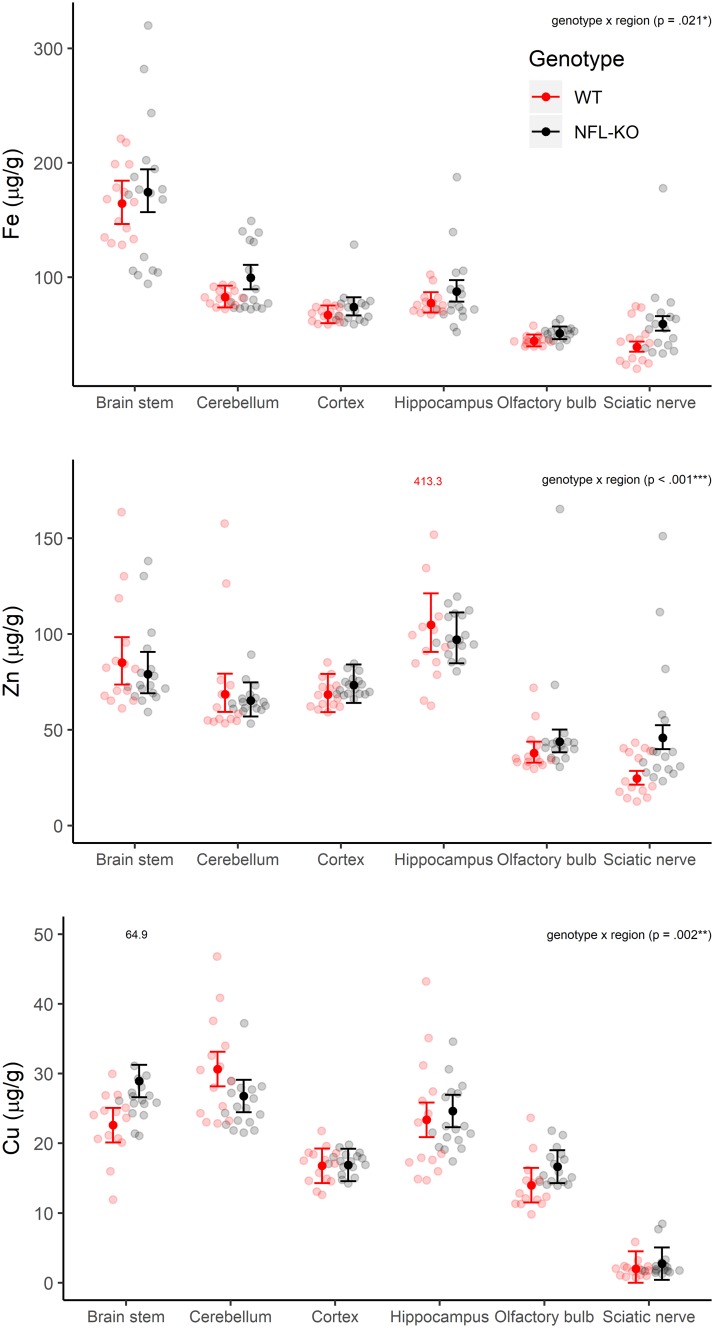
Metal variability in different brain regions. Estimated marginal means (and 95% CI) show patterns of Fe, Zn, and Cu composition in different brain regions for the two genotypes (WT and NFL-KO; averaged over 5mo and 22mo animals). There were significant genotype x region interactions for Zn, Fe and Cu (Zn: F_(5, 140)_ = 8, *p* < .001; Fe: F_(5, 140)_ = 3, *p* = .021; F_(5, 139)_ = 4, *p* = .002).

Genotype x age (Fig 1) and genotype x region (Fig 2) interactions were investigated for each metal cation in a model which accounted for region x age interactions and intra-class correlation within region for each animal. For Zn and Fe, there were significant genotype x age interactions (Zn: F_(1, 27)_ = 9, *p* = .005; Fe: F_(1, 27)_ = 11, *p* = .002) and genotype x region interactions (Zn: F_(5, 140)_ = 8, *p* < .001; Fe: F_(5, 140)_ = 3, *p* = .021). For Cu, only the genotype x region interaction was significant (F_(5, 139)_ = 4, *p* = .002).

The standardized effect of genotype on Cu accumulation in tissues was small ($f_{Cu}^{2}=0.09$), however the standardized effect of genotype on Zn and Fe accumulation in tissues was moderate ($f_{log(Zn)}^{2}=0.27,f_{log(Fe)}^{2}=0.25$).

The current study shows that there were differences in Cu, Zn and Fe concentrations across regions of the nervous system. For example, Fe was relatively abundant in the brain stem, with lower levels in the olfactory bulb. The olfactory bulb also showed relatively lower levels of both Zn and Cu. The sciatic nerve demonstrated very low levels of Cu, relative to Fe and Zn.

One of the most prominent differences between NFL KO and WT mice was found for Fe, especially for older animals. Moderate genotype differences were also detected for Zn, with the most pronounced difference was present for sciatic nerve. In many regions, Zn abundance did not decrease in the NFL KO mice over ageing relative to the WT mice. Consistent patterns of genotype difference for Cu were not observed.

These studies were motivated by our previous observations that APPswe/PS1dE9 mice on a NFL KO background demonstrated higher amyloid plaque deposition [15]. In this regard, we were interested in potential differences in brain content of Fe, Zn and Cu, metals implicated in beta amyloid processing and aggregation into plaques, that may be related to the absence of NFL and a complete neurofilamentous network [16,17]. The relatively increased levels of Fe across most brain regions of aged NFL KO mice, as well as relatively maintained levels of Zn, may be particularly significant in the context of contributing to an environment that drives Alzheimer’s disease pathology. We have previously shown that there are substantial changes in NF localisation in the hippocampus during ageing, that abnormal accumulation of NFs in dystrophic neurites surrounding plaques is the earliest neuronal pathology of AD and that tau pathology replaces the normal NF network in neurofibrillary tangles and dystrophic neurites [13]. Changes to the integrity of neurofilamentous networks during ageing could possibly also follow decades of wear and tear on axons, and may possibly result in increased levels of Fe in the extracellular environment, which then impact on multiple aspects of AD pathogenesis. The translation of APP, which is the parent protein to the Abeta of extracellular plaques in the AD brain, is regulated by an iron response element in the APP mRNA [19,20] and the secretase cleavage of APP to form Abeta is also regulated by iron. Furthermore, the aggregation and oligomerization of Abeta is potentiated by iron, which is also likely to contribute to Abeta-mediated oxidative damage [21–26]. Similarly, iron can induce the aggregation of tau [27] and the association of redox active iron with tau and neurofibrillary tangles may contribute to oxidative stress [28]. In addition, a loss of NFs in substantia nigra neurons in the early stages of PD [11] may also be linked to the accumulation of Fe in these cells [1].

The current data supports the existence of a relationship between NF protein levels and metal ions in the brain, potentially relating to the regulation and/or storage of metals. Cations may also have direct roles in the organisation of neurofilamentous networks. Iron deficiency, for example, can impact neurofilament phosphorylation during development [29]. Copper and iron may also have a role in catechol-mediated cross-linking of neurofilament proteins [30], and copper and zinc have the capacity to directly bind to the NFH subunit [31]. Divalent ions such as Ca, Mg and Zn have been shown to act as cross-linkers between NFs, influencing the gelation and viscoelastic properties of neurofilamentous networks [32,33]. Finally, atomic force microscopy experiments support the cross-linking role of divalent cations, particularly in moderating the spacing and mechanical properties of phosphorylated NF side-arm structures [34].

## Conclusions

Cumulatively, these data show that the absence of NF-L, and a neurofilamentous network perturbs the processing and/or storage of Fe through ageing, leading to higher levels of this metal in the brain and sciatic nerve. Biomarker studies indicate a progressive perturbation of NFs with ageing and neuroaxonal degeneration involving these proteins across a wide range of neurodegenerative conditions [35]. Hence, alterations in neurofilaments during brain ageing may, thus, create an intracellular or extracellular environment of excess Fe, which could contribute to risk of either neuronal degeneration or protein accumulation in neurodegenerative conditions such as Alzheimer’s and/or Parkinson’s disease.
